# The Glasgow Prognostic Score Before Curative Resection May Predict Postoperative Complications in Patients with Gastric Cancer

**DOI:** 10.1007/s12029-021-00689-9

**Published:** 2021-09-14

**Authors:** Yota Shimoda, Hirohito Fujikawa, Keisuke Komori, Hayato Watanabe, Kosuke Takahashi, Kazuki Kano, Takanobu Yamada, Manabu Shiozawa, Soichiro Morinaga, Kenji Katsumata, Akihiko Tsuchida, Takashi Ogata, Takashi Oshima

**Affiliations:** 1 Department of Gastrointestinal Surgery, Kanagawa Cancer Center, 2-3-2 Nakao, Asahi-ku, Yokohama, Japan; 2 Department of Gastrointestinal and Pediatric Surgery, Tokyo Medical University, 6-1-1 Shinjuku, Shinjuku-ku, Tokyo, Japan

**Keywords:** Gastric cancer, Glasgow prognostic score, Postoperative complications

## Abstract

**Purpose:**

Despite improvements in surgical techniques and devices and perioperative care of gastric cancer (GC), the rate of postoperative complications still has not decreased. If patients at high risk for postoperative complications could be identified early using biomarkers, these complications might be reduced. In this study, we investigated usefulness of the preoperative Glasgow Prognostic Score (GPS) as a predictive factor for complications after surgery in patients with stage II/III GC.

**Methods:**

This study retrospectively analyzed the outcomes of 424 patients who underwent curative surgery for pathological stage II/III GC from February 2007 to July 2019 at a single center. The GPS was assessed within 4 days before surgery. To identify independent risk factors for postoperative complications, univariate and multivariate analyses were performed using a Cox proportional hazards model.

**Results:**

The numbers of patients with a GPS of 0, 1, and 2 were 357, 55, and 12, respectively. The rate of complications after surgery was significantly higher among patients with a GPS of 1 or 2 than among patients with a GPS of 0 (*p* = 0.008). Multivariate analysis identified a GPS of 1 or 2 as an independent predictive factor for postoperative complications (*p* = 0.037).

**Conclusion:**

The preoperative GPS may be a useful predictive factor for postoperative complications in patients with stage II/III GC. Being aware of the risk of complications after surgery as indicated by the GPS before surgery may promote safe and minimally invasive surgery that we expect will improve outcomes in patients with a GPS of 1 or 2.

## Introduction

Gastric cancer (GC) is the fifth most prevalent carcinoma in the world, with 1,089,103 new cases in 2020, and the fourth leading cause of death from cancer, with 768,793 deaths globally [1]. The standard treatment for pathological (p)stage II/III GC is curative surgery and adjuvant chemotherapy [2].

Despite improvements in surgical techniques and devices and perioperative care, the rate of postoperative complications still has not decreased. Recent studies reported that postoperative complication rates after GC resection were 17.4–24.5% [3–5]. Postoperative complications may significantly impact both long-term and short-term outcomes because they can sometimes lead to the production of cytokines that are growth factors for GC micrometastases. Complications prolong hospital stays, reduce the quality of life of patients, and increase health care costs [6, 7]. Additionally, postoperative complications often delay the initiation of adjuvant chemotherapy and decrease patients’ tolerance of chemotherapy [8]. Furthermore, a postoperative systemic inflammatory response is related to poor cancer-specific survival independent of the tumor stage [9]. Postoperative complications contribute to poor cancer-specific survival in various types of cancer including GC [10–12]. Therefore, if patients at high risk for postoperative complications could be identified early using biomarkers, these complications might be reduced by selecting risk-adapted procedures and perioperative management. Such biomarkers must be easy-to-use, low-cost, rapid, and objective measures accessible to all patients and hospitals.

The Glasgow Prognostic Score (GPS) has been reported as a parameter that elevated serum C-reactive protein (CRP) levels reflect a progression cancer stage, and decreased serum albumin levels are an indicator of malnutrition [13–18]. Therefore, we hypothesized that the GPS might be a useful predictor of postoperative complications. In this study, we investigated the usefulness of the GPS before surgery in predicting complications after surgery in patients with stage II/III GC.

## Patients and Methods

### Patients

The study was approved by the Research Ethics Committee of the Kanagawa Cancer Center in Yokohama, Japan, before the study started (approval number: Epidemiological Study, 2019 − 113). A total of 623 patients who underwent gastrectomy with D2 lymph node dissection for pstage II/III GC from February 2007 to July 2019 at the Kanagawa Cancer Center measured serum albumin and CRP levels before surgery and documented in their medical record were eligible for this study. Of those patients, patients who underwent preoperative treatment including neoadjuvant chemotherapy, those with remnant GC, those with stage IV GC, and those with non-curative (R1 or R2) resection were excluded. Finally, a total of 424 patients were analyzed.

### Definition of GPS

The GPS was calculated using the serum CRP and serum albumin levels extracted from the medical records. Serum albumin and CRP levels before surgery were assessed within 4 days before surgery. The GPS was scored by allocating one point each for hypoalbuminemia (< 3.5 mg/dL) and elevated CRP (> 1.0 mg/dL). Patients with a hypoalbuminemia (< 3.5 mg/dL) and elevated CRP (> 1.0 mg/dL) were assigned a score of 2. Those with a hypoalbuminemia alone or elevated CRP alone were assigned a score of 1. Those with normal albumin (≥ 3.5 mg/dL) and CRP (CRP ≤ 1.0 mg/dL) levels were assigned a score of 0 [19].

### Surgical Procedure and Perioperative Care

Patient with cstage IB GC underwent laparoscopy-assisted gastrectomy with D1 + lymphadenectomy, and those with cstage II/III GC underwent open gastrectomy with D2 lymphadenectomy according to the TNM classification (8th edition).

Our center uses the “enhanced recovery after surgery” protocol, which has been described in a previous study [20]. Oral intake was initiated on postoperative day (POD) 1, beginning with water. Patients began to eat on POD 2, starting with rice gruel and advancing in three steps to regular food intake on POD 6.

### Data Collection

All variables, including patient age, sex, body mass index (kg/m^2^), type of surgery, operative time, blood loss, depth of invasion, and lymph node metastasis, were collected from the clinicopathological database in Kanagawa Cancer Center. All resected specimens had been examined and histopathologically staged according to UICC TNM 8th edition [21]. Complications after surgery were defined as those observed within 1 month after surgery that were grade 2 or higher according to the Clavien-Dindo classification [22].

### Evaluations and Statistical Analysis

Patients were divided into a GPS 0 group and a GPS 1 or 2 group based on their preoperative GPS. We used the Mann–Whitney *U* test for comparison of age, body mass index, operation time, and intraoperative blood loss between two groups. Categorical variables were analyzed using Pearson’s *χ*^2^ test. To identify independent risk factors for postoperative complications, univariate and multivariate analyses were performed using a Cox proportional hazards model. All statistical analyses were performed using IBM SPSS Statistics for Windows, version 24.0 (IBM Corp., Armonk, NY, USA). Statistical significance was defined at a *p*-value < 0.05.

## Results

### Comparison of Clinicopathological Characteristics Between the GPS 0 and GPS 1 or 2 Groups

**Fig. 1:**
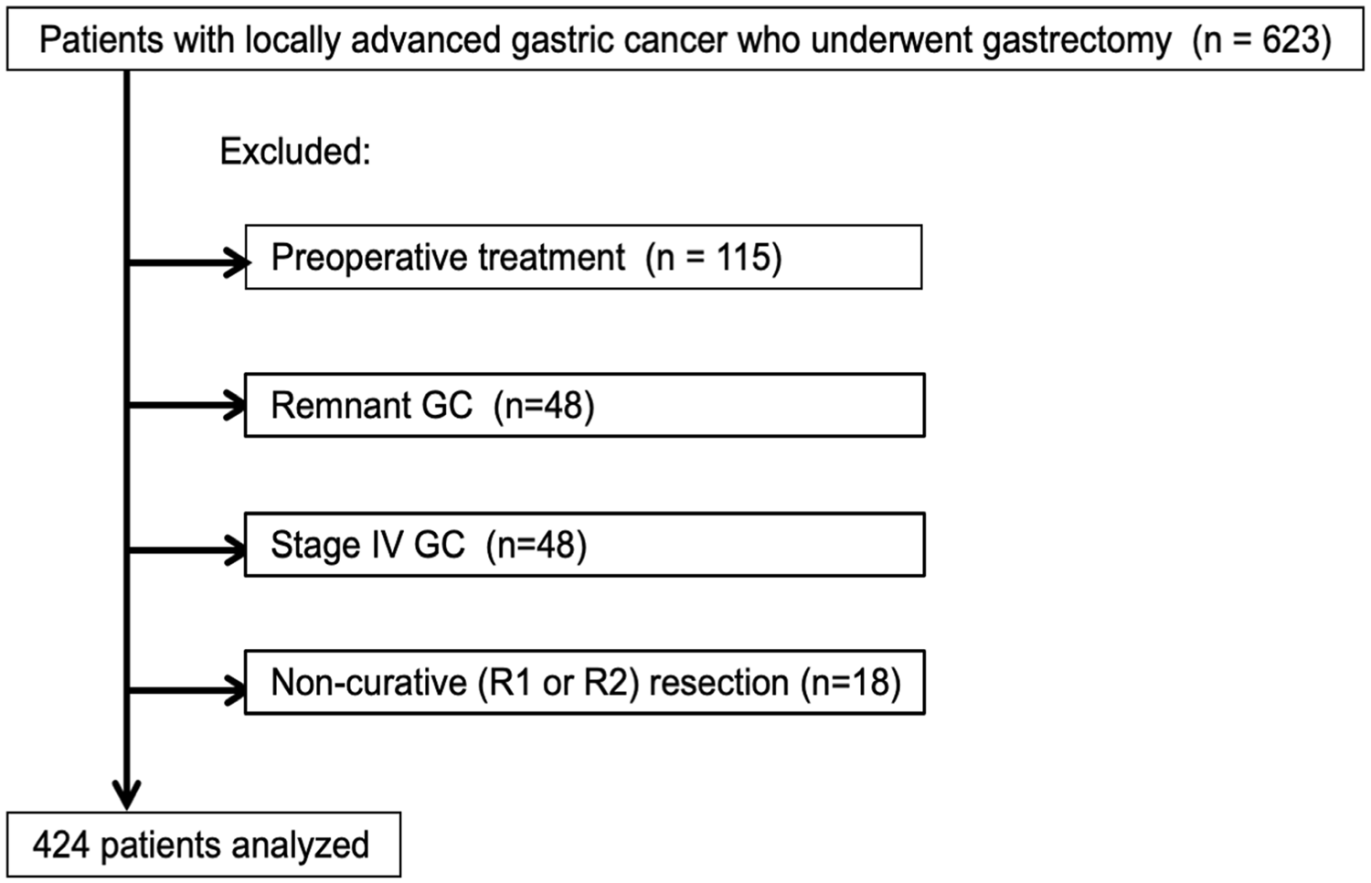
Flow diagram of patient selection in this retrospective single-center study of gastric cancer patients. A total of 623 consecutive patients who underwent gastrectomy with D2 lymph node dissection for pStage II/III GC from February 2007 to July 2019 at the Kanagawa Cancer Center and measured serum albumin and CRP levels before surgery were eligible for this study. Of those patients, patients who underwent preoperative treatment including neoadjuvant chemotherapy, those with remnant GC, those with stage IV GC, and those with non-curative (R1 or R2) resection were excluded. Finally, a total of 424 patients were analyzed

In this study, a total of 424 patients were examined. A flow diagram of the patient selection is shown in Fig. 1. The numbers of patients with a GPS of 0, 1, and 2 were 357, 55, and 12, respectively. The clinicopathological characteristics of the GPS 0 and GPS 1 or 2 groups are shown in Table 1. Age, operative time, blood loss during surgery, and pathological lymph node metastasis were significantly higher in the GPS 1 or 2 group than in the GPS 0 group. The body mass index was significantly lower in the GPS 1or 2 group than in the GPS 0 group.

**Table 1:** Clinicopathological characteristics of patients in the GPS 0 and GPS 1 or 2 groups

	GPS		
Variables/categories	0 (*n* = 357)	1 or 2 (*n* = 67)	*p*-Value
Age, years (mean ± SD)	66.7 ± 11.0	72.2 ± 8.8	** < 0.001**
Sex, *n* (%)			
Male	239 (67)	43 (64)	0.66
Female	118 (33)	24 (36)	
Body mass index, kg/m^2^ (mean ± SD)	22.7 ± 3.1	21.8 ± 3.1	**0.035**
Type of surgery, *n* (%)			
Distal gastrectomy	199 (56)	30 (45)	0.098
Total gastrectomy	158 (44)	37 (55)	
Operation time, min (mean ± SD)	214.6 ± 63.6	238.8 ± 71.5	**0.005**
Blood loss, mL (mean ± SD)	335.6 ± 334.9	497.4 ± 404.5	**0.001**
Pathological tumor depth, *n* (%)			
T2	98 (27)	12 (18)	0.157
T3	104 (29)	26 (39)	
T4	155 (44)	29 (43)	
Pathological lymph node metastasis, *n* (%)			
N0	135 (38)	14 (21)	**0.038**
N1	71 (20)	13 (19)	
N2	65 (18)	17 (25)	
N3	86 (24)	23 (34)	

*GPS* Glasgow Prognostic Score, *SD* standard deviation

### Comparison of Postoperative Complications Between the GPS 0 and GPS 1 or 2 Groups

Sixty-six postoperative complications (18%) were observed in the GPS 0 group and 22 (33%) in the GPS 1 or 2 group. The rate of postoperative complications was significantly higher in the GPS 1 or 2 group than in the GPS 0 group (*p* = 0.008). The postoperative complications in the two groups are shown in Table 2. The rate of infectious complications, such as pancreatic fistula, anastomotic leakage, pneumonia, intraabdominal abscess, and wound infection, was significantly higher in the GPS 1 or 2 group than in the GPS 0 group (*p* = 0.008).

**Table 2:** Comparison of postoperative complications between the GPS 0 and GPS 1 or 2 groups

	GPS		
Variables/categories, *n* (%)	0 (*n* = 357)	1 or 2 (*n* = 67)	*p*-Value
All postoperative complications	66 (18)	22 (33)	**0.008**
Pancreatic fistula	21 (6)	6 (9)	0.345
Anastomotic leakage	12 (3)	4 (6)	0.304
Ileus	7 (2)	3 (4)	0.213
Pneumonia	5 (1)	4 (6)	**0.017**
Intraabdominal abscess	5 (1)	1 (1)	0.953
Postoperative bleeding	4 (1)	1 (1)	0.796
Wound infection	3 (1)	0 (0)	0.451
Delayed gastric emptying	2 (1)	0 (0)	0.539
Others	9 (3)	2 (2)	0.826
Infectious complication	45 (13)	16 (24)	**0.016**
Noninfectious complication	21 (6)	6 (9)	0.345

*GPS* Glasgow Prognostic Score

### Risk Factors of Postoperative Complication

Table 3 shows the results of the univariate analysis of postoperative complications. The types of the surgical procedure, operative time, blood loss, and GPS were significantly associated with postoperative complications. Table 4 shows the results of the multivariate logistic regression analysis. An operation time ≥ 200 min (hazard ratio [HR] 1.947, 95% confidence interval [CI] 1.080 − 3.512, *p* = 0.027), and a GPS of 1 or 2 (HR 1.877, 95% CI 1.039 − 3.388, *p* = 0.037) was identified as independent risk factors for postoperative complications.

**Table 3:** Univariate analysis of variables and their potential relation to the occurrence of postoperative complications

Variables/categories	Patients (*n*)	Complications *n* (%)	*p*-Value
Age, years			
< 75	306	60 (20)	0.348
≥ 75	118	28 (24)	
Sex			
Male	282	66 (23)	0.058
Female	142	22 (15)	
Body mass index, kg/m^2^			
< 25	345	71 (21)	0.853
≥ 25	79	17 (22)	
Surgical procedure			
Distal gastrectomy	229	36 (16)	**0.006**
Total gastrectomy	195	52 (27)	
Operation time, min			
< 200	179	23 (13)	**0.001**
≥ 200	245	65 (27)	
Blood loss, mL			
< 250	202	32 (16)	**0.017**
≥ 250	222	56 (25)	
Pathological tumor depth			
2 and 3	241	55 (23)	0.228
4	183	33 (18)	
Pathological lymph node metastasis			
( −)	149	33 (22)	0.175
( +)	275	55 (20)	
Glasgow Prognostic Score			
0	357	66 (18)	**0.008**
1 and 2	67	22 (33)	

*GPS* Glasgow Prognostic Score

**Table 4:** Multivariate analysis of selected variables and their relation to the occurrence of postoperative complications

	OR	95% CI	*p*-Value
Surgical procedure			
Distal gastrectomy			
Total gastrectomy	1.579	0.958–2.601	0.073
Operation time, min			
< 200			
≥ 200	1.947	1.080–3.512	**0.027**
Blood loss, mL			
< 250			
≥ 250	1.947	1.080–3.512	0.584
GPS			
0			
1 and 2	1.877	1.039–3.388	**0.037**

*OR* odds ratio, *CI* confidence interval, *GPS* Glasgow Prognostic Score

## Discussion

In the present study, we investigated the usefulness of the GPS calculated using the serum CRP and serum albumin levels before surgery in predicting postoperative complications in patients with stage II/III GC who underwent curative surgery. Our results confirm our hypothesis that the preoperative GPS is an independent predictive marker of postoperative complications.

Today, the most commonly used clinical biomarker of systemic inflammation is the serum CRP level. There is an association between systemic inflammation and complications after oncologic surgery [23, 24]. It has been reported that proinflammatory cytokines such as interleukin-1, interleukin-6, and tumor necrosis factor-α induce an increase in serum CRP levels [25]. Those cytokines produced by various cancers and lymphocytes sensitized to cancer cause systemic inflammatory responses and cancer cachexia [26]. Preoperatively elevated serum CRP levels are associated with an increased incidence of postoperative complications in cancer patients [27], while serum albumin is produced in the liver and is the most abundant serum protein [28]. Low albumin is an indicator of malnutrition, and preoperative hypoalbuminemia is often seen in patients with advanced GC. Serum albumin levels decline in patients with poor nutritional status, loss of skeletal muscle, and systemic inflammatory response [29]. In cancer, the progression of the disease is associated with increasing systemic inflammation, leading to hyper catabolism and decreased serum albumin levels [30]. Several studies have demonstrated that hypoalbuminemia increases the incidence of postoperative complications in cancer patients [31–33].

We hypothesized that the GPS could be a useful predictor of postoperative complications in patients with GC because it is calculated based on serum CRP and albumin levels. However, until now, few studies have examined the association between the GPS and postoperative complications. As for previous reports, Fujiwara et al. [34] showed that the GPS was related to blood transfusion requirements and postoperative complications in patients with hepatocellular carcinoma undergoing resection. Moyes et al. [35] reported that a preoperative elevated modified GPS was independently associated with an increased risk of postoperative infectious complications in patients undergoing resection of colorectal cancer. As for the study in patients with GC, Kubota et al. [36] did not find an association between the preoperative GPS and the occurrence of postoperative complications after curative resection of GC. While, in our study, the preoperative GPS was identified as a predictor of postoperative complications in patients with GC. The rate of total gastrectomy in the GPS 1 or 2 group in our study was 55%, which was more than twice that in their study. In general, because complications of total gastrectomy, such as anastomotic insufficiency, are higher than those of subtotal gastrectomy, this difference might be explained by the different proportions of total gastrectomy to subtotal gastrectomy in the two studies.

As for possible clinical application of the preoperative GPS, because the preoperative GPS may identify patients with a high risk of postoperative complications, it may assist surgeons in assessing the risk of postoperative complications and choosing an approach that is as safe and minimally invasive as possible. Furthermore, recent studies have shown the efficacy of perioperative immune-nutritional support according to the condition of each cancer patient in reducing the incidence of postoperative complications [37, 38]. In patients with GPS 1, those with low serum albumin and CRP levels may be undernutrition related with cancer, while those with high serum albumin and CRP levels may be pre-cachexic. Patients of GPS 2 with low serum albumin and high CRP levels may be cancer cachexia. It was reported that supportive nutritional interventions in patients with preoperative cancer cachexia and pre-cachexic may be ineffective and early supportive nutritional interventions for patients with undernutrition related with cancer can be effective for improvement of preoperative nutritional status [37, 38].

Our study had a limitation. Our study was retrospective study in a single affiliation. Prospective and multicenter studies in larger cohorts are necessary to clarify the predictive value of preoperative GPS for postoperative complications in GC patients.

In conclusion, the GPS before surgery may be a useful predictive marker for complications after surgery in patients with stage II/III GC who underwent curative resection. Being aware of the risk of complications after surgery as indicated by the GPS before surgery may promote safe and minimally invasive surgery that we expect will improve outcomes in these patients.

## Data Availability

A total of 623 patients who underwent gastrectomy with D2 lymph node dissection for pstage II/III GC from February 2007 to July 2019 at the Kanagawa Cancer Center, measured serum albumin and CRP levels before surgery, and documented in their medical record were eligible for this study.

## References

[1] International Agency for Research on Cancer: The Global Cancer Observatory. (2020) https://gco.iarc.fr/today/data/factsheets/cancers/7-Stomach-fact-sheet.pdf. Accessed 29 April 2021.

[2] Sasako M, Sakuramoto S, Katai H, Kinoshita T, Furukawa H, Yamaguchi T, Nashimoto A, Fujii M, Nakajima T, Ohashi Y (2011). Five-year outcomes of a randomized phase III trial comparing adjuvant chemotherapy with S-1 versus surgery alone in stage II or III gastric cancer. J Clin Oncol.

[3] Kurita N, Miyata H, Gotoh M, Shimada M, Imura S, Kimura W, Tomita N, Baba H, Kitagawa Y, Sugihara K, Mori M (2015). Risk model for distal gastrectomy when treating gastric cancer on the basis of data from 33,917 Japanese patients collected using a nationwide web-based data entry system. Ann Surg.

[4] Katai H, Sasako M, Fukuda H, Nakamura K, Hiki N, Saka M, Yamaue H, Yoshikawa T, Kojima K, Takagane A, Fukushima N, Katai H, Saka M, Kojima K, Inokuchi M, Yamada H, Hiki N, Fukunaga T, Yoshiba H, Tokunaga M, Yoshikawa T, Cho H, Mochizuki Y, Misawa K, Uyama I, Kanaya S, Taniguchi K, Imamoto H, Miyashiro I, Tanigawa N, Iwahashi M, Takifuji K, Nishizaki M, Kitanov S, Shiraishi N, Eto T (2010). Safety and feasibility of laparoscopy-assisted distal gastrectomy with suprapancreatic nodal dissection for clinical stage I gastric cancer: a multicenter phase II trial (JCOG 0703). Gastric Cancer.

[5] Kanda M, Kobayashi D, Tanaka C, Iwata N, Yamada S, Fujii T, Nakayama G, Sugimoto H, Koike M, Nomoto S, Murotani K, Fujiwara M, Kodera Y (2016). Adverse prognostic impact of perioperative allogeneic transfusion on patients with stage II/III gastric cancer. Gastric Cancer.

[6] Shigeishi H, Ohta K, Takechi M (2015). Risk factors for postoperative complications following oral surgery. J Appl Oral Sci.

[7] Chauhan A, House MG, Pitt HA, Nakeeb A, Howard TJ, Zyromski NJ, Schmidt CM, Ball CG, Lillemoe KD (2011). Post-operative morbidity results in decreased long-term survival after resection for hilar cholangiocarcinoma. HPB (Oxford).

[8] Jin LX, Sanford DE, Squires MH, Moses LE, Yan Y, Poultsides GA, Votanopoulos KI, Weber SM, Bloomston M, Pawlik TM, Hawkins WG, Linehan DC, Schmidt C, Worhunsky DJ, Acher AW, Cardona K, Cho CS, Kooby DA, Levine EA, Winslow E, Saunders N, Spolverato G, Colditz GA, Maithel SK, Fields RC (2016). Interaction of postoperative morbidity and receipt of adjuvant therapy on long-term survival after resection for gastric adenocarcinoma: results from the U.S. Gastric Cancer Collaborative Ann Surg Oncol.

[9] McArdle CS, McMillan DC, Hole DJ (2005). Impact of anastomotic leakage on long-term survival of patients undergoing curative resection for colorectal cancer. Br J Surg.

[10] Khuri SF, Henderson WG, DePalma RG, Mosca C, Healey NA, Kumbhani DJ, Participants in the VA National Surgical Quality Improvement Program. Determinants of long-term survival after major surgery and the adverse effect of postoperative complications. Ann Surg. 2005;242:326–41; discussion 341–3. 10.1097/01.sla.0000179621.33268.83 10.1097/01.sla.0000179621.33268.83 PMC1357741 16135919

[11] Kusano T, Sasaki A, Kai S, Endo Y, Iwaki K, Shibata K, Ohta M, Kitano S (2009). Predictors and prognostic significance of operative complications in patients with hepatocellular carcinoma who underwent hepatic resection. Eur J Surg Oncol.

[12] Watanabe H, Hayashi T, Komori K, Hara K, Maezawa Y, Kano K, Shimoda Y, Fujikawa H, Aoyama T, Yamada T, Yamamoto N, Cho H, Ito H, Shiozawa M, Yukawa N, Morinaga S, Yoshikawa T, Rino Y, Masuda M, Ogata T, Oshima T. Impact of postoperative complications on recurrence in patients with stage II/III gastric cancer who received adjuvant chemotherapy with S-1. Anticancer Res. 2020;40:1683–1690. 10.21873/anticanres.14120 10.21873/anticanres.14120 32132075

[13] Kasahara N, Sunaga N, Tsukagoshi Y, Miura Y, Sakurai R, Kitahara S, Yokobori T, Kaira K, Mogi A, Maeno T, Asao T, Hisada T. Post-treatment Glasgow Prognostic Score predicts efficacy in advanced non-small-cell lung cancer treated with anti-PD1. Anticancer Res. 2019;39:1455–1461. 10.21873/anticanres.13262 10.21873/anticanres.13262 30842182

[14] Shiba H, Misawa T, Fujiwara Y, Futagawa Y, Furukawa K, Haruki K, Iwase R, Wakiyama S, Ishida Y, Yanaga K (2013). Glasgow prognostic score predicts therapeutic outcome after pancreaticoduodenectomy for carcinoma of the ampulla of Vater. Anticancer Res.

[15] Yamada S, Fujii T, Yabusaki N, Murotani K, Iwata N, Kanda M, Tanaka C, Nakayama G, Sugimoto H, Koike M, Fujiwara M, Kodera Y (2016). Clinical implication of inflammation-based prognostic score in pancreatic cancer: Glasgow prognostic score is the most reliable parameter. Medicine (Baltimore).

[16] Li MX, Bi XY, Li ZY, Huang Z, Han Y, Zhou JG, Zhao JJ, Zhang YF, Zhao H, Cai JQ (2015). Prognostic role of Glasgow prognostic score in patients with hepatocellular carcinoma: a systematic review and meta-analysis. Medicine (Baltimore).

[17] Dréanic J, Maillet M, Dhooge M, Mir O, Brezault C, Goldwasser F, Chaussade S, Coriat R (2013). Prognostic value of the Glasgow Prognostic Score in metastatic colorectal cancer in the era of anti-EGFR therapies. Med Oncol.

[18] Inamoto T, Matsuyama H, Sakano S, Ibuki N, Takahara K, Komura K, Takai T, Tsujino T, Yoshikawa Y, Minami K, Nagao K, Inoue R, Azuma H. The systemic inflammation-based Glasgow Prognostic Score as a powerful prognostic factor in patients with upper tract urothelial carcinoma. Oncotarget. 2017;8:113248–113257. 10.18632/oncotarget.22641 10.18632/oncotarget.22641 PMC5762588 29348903

[19] Forrest LM, McMillan DC, McArdle CS, Angerson WJ, Dunlop DJ (2003). Evaluation of cumulative prognostic scores based on the systemic inflammatory response in patients with inoperable non-small-cell lung cancer. Br J Cancer.

[20] Yamada T, Hayashi T, Aoyama T, Shirai J, Fujikawa H, Cho H, Yoshikawa T, Rino Y, Masuda M, Taniguchi H, Fukushima R, Tsuburaya A (2014). Feasibility of enhanced recovery after surgery in gastric surgery: a retrospective study. BMC Surg.

[21] Brierley JD, Gospodarowicz MK, Wittekind C, Editors. TNM Classification of Malignant Tumors. 2017;8th ed. ISBN:978–1–119–26357–9, West Sussex: Wiley-Blackwell.

[22] Clavien PA, Barkun J, de Oliveira ML, Vauthey JN, Dindo D, Schulick RD, de Santibañes E, Pekolj J, Slankamenac K, Bassi C, Graf R, Vonlanthen R, Padbury R, Cameron JL, Makuuchi M (2009). The Clavien-Dindo classification of surgical complications: five-year experience. Ann Surg.

[23] Mahmoud FA, Rivera NI (2002). The role of C-reactive protein as a prognostic indicator in advanced cancer. Curr Oncol Rep.

[24] Coussens LM, Werb Z (2002). Inflammation and cancer. Nature.

[25] Lopez-Pastorini A, Riedel R, Koryllos A, Beckers F, Ludwig C, Stoelben E (2017). The impact of preoperative elevated serum C-reactive protein on postoperative morbidity and mortality after anatomic resection for lung cancer. Lung Cancer.

[26] Dutta S, Al-Mrabt NM, Fullarton GM, Horgan PG, McMillan DC (2011). A comparison of POSSUM and GPS models in the prediction of post-operative outcome in patients undergoing oesophago-gastric cancer resection. Ann Surg Oncol.

[27] Selby J, Prabhudesai A (2014). Can C-reactive protein predict the severity of a post-operative complication after elective resection of colorectal cancer?. Int J Colorectal Dis.

[28] Fuhrman MP (2002). The albumin-nutrition connection: separating myth from fact. Nutrition.

[29] Gabay C, Kushner I (1999). Acute-phase proteins and other systemic responses to inflammation. N Engl J Med.

[30] Oñate-Ocaña LF, Aiello-Crocifoglio V, Gallardo-Rincón D, Herrera-Goepfert R, Brom-Valladares R, Carrillo JF, Cervera E, Mohar-Betancourt A (2007). Serum albumin as a significant prognostic factor for patients with gastric carcinoma. Ann Surg Oncol.

[31] Leung JS, Seto A, Li GK (2017). Association between preoperative nutritional status and postoperative outcome in head and neck cancer patients. Nutr Cancer.

[32] Caras RJ, Lustik MB, Kern SQ, McMann LP, Sterbis JR (2017). Preoperative albumin is predictive of early postoperative morbidity and mortality in common urologic oncologic surgeries. Clin Genitourin Cancer.

[33] Chen Y, Wu G, Wang R, Chen J (2018). Preoperative albumin level serves as a predictor for postoperative pulmonary complications following elective laparoscopic gastrectomy. Curr Pharm Des.

[34] Fujiwara Y, Shiba H, Furukawa K, Iida T, Haruki K, Gocho T, Wakiyama S, Hirohara S, Ishida Y, Misawa T, Ohashi T, Yanaga K (2010). Glasgow Prognostic Score is related to blood transfusion requirements and post-operative complications in hepatic resection for hepatocellular carcinoma. Anticancer Res.

[35] Moyes LH, Leitch EF, McKee RF, Anderson JH, Horgan PG, McMillan DC (2009). Preoperative systemic inflammation predicts postoperative infectious complications in patients undergoing curative resection for colorectal cancer. Br J Cancer.

[36] Kubota T, Hiki N, Nunobe S, Kumagai K, Aikou S, Watanabe R, Sano T, Yamaguchi T (2012). Significance of the inflammation-based Glasgow Prognostic Score for short- and long-term outcomes after curative resection of gastric cancer. J Gastrointest Surg.

[37] Kanekiyo S, Takeda S, Iida M, Nishiyama M, Kitahara M, Shindo Y, Tokumitsu Y, Tomochika S, Tsunedomi R, Suzuki N, Abe T, Yoshino S, Hazama S, Ueno T, Nagano H (2019). Efficacy of perioperative immunonutrition in esophageal cancer patients undergoing esophagectomy. Nutrition.

[38] Zhang X, Chen X, Yang J, Hu Y, Li K (2020). Effects of nutritional support on the clinical outcomes of well-nourished patients with cancer: a meta-analysis. Eur J Clin Nutr.

